# Perceived Interpersonal Burdensomeness as a Mediator between Nightmare Distress and Suicidal Ideation in Nightmare Sufferers

**DOI:** 10.3389/fpsyg.2016.01805

**Published:** 2016-11-18

**Authors:** Sooyeon Suh, Matthew Schneider, Ruda Lee, Thomas Joiner

**Affiliations:** ^1^Department of Psychology, Sungshin Women’s UniversitySeoul, South Korea; ^2^Department of Psychiatry, Stanford UniversityPalo Alto, CA, USA; ^3^Department of Psychology, Florida State UniversityTallahassee, FL, USA

**Keywords:** nightmares, perceived burdensomeness, suicidal ideation, interpersonal theory of suicide, Republic of Korea

## Abstract

Previous studies have supported the significant association between nightmares and suicidal ideation, but the underlying mechanisms are largely unknown. The purpose of the present study was to investigate perceived burdensomeness and thwarted belongingness as mediators in the relationship between nightmare distress and suicidal ideation. This sample consisted of 301 undergraduate students who endorsed experiencing nightmares (mean age 21.87 ± 2.17, 78.1% female). All participants completed questionnaires on nightmare distress (Nightmare Distress Questionnaire), unmet interpersonal needs (Interpersonal Needs Questionnaire), and suicidal ideation (Depressive Symptom Inventory – Suicidality Subscale). Analyses were performed using multiple mediation regression. Results indicated that nightmare distress was associated with perceived burdensomeness (*r* = 0.17, *p* < 0.001) and suicidal ideation (*r* = 0.24, *p* < 0.001), but was not related to thwarted belongingness (*r* = 0.10, *p* = 0.06). Multiple mediation analyses revealed that perceived burdensomeness partially mediated the relationship between nightmares and suicidal ideation, but thwarted belongingness did not. Additionally, this mediating relationship for perceived burdensomeness was moderated by gender, being significant only for females. These findings highlight the important role of interpersonal factors in the relationship between nightmares and suicidal ideation.

## Introduction

Nightmares are vivid and very disturbing dreams in which unpleasant and intensified emotion awaken the sleeper (Levin and Fireman, 2002). Previous studies support the strong association between nightmares and suicidal behavior, independent of insomnia, depression, anxiety, and Post-Traumatic Stress Disorder (PTSD) (Nadorff et al., 2011). In a study with suicide attempters, individuals with frequent nightmares had three times higher risk for repeated suicide attempts compared to individuals with infrequent nightmares (Sjostrom et al., 2009). In another study, among different sleep variables such as difficulty initiating or maintaining sleep, nightmares had the highest association with suicidality in suicide attempters after adjusting for psychiatric diagnoses and psychiatric symptom intensity (Sjostrom et al., 2007). Although nightmares have traditionally been viewed as a risk factor for various forms of suicidality, the underlying mechanisms between nightmares and suicidal outcomes, especially in the context of interpersonal factors, has not been closely examined.

It has been suggested that frequent nightmares may exacerbate feelings of ineffectiveness (Bernert and Joiner, 2007). Sleep disturbance and poor sleep quality may affect the ability to regulate emotions and thus respond adequately in interpersonal relationships (Nielsen and Lara-Carrasco, 2007), although few studies have investigated the effect of nightmares on interpersonal functioning. One study found that individuals with frequent nightmares (more than three times in 3 weeks) had a significantly higher score on the interpersonal sensitivity subscale of the Symptoms Checklist-90 questionnaire compared to those with medium (1–2 times/3 weeks) or low frequency (0 times/3 weeks) nightmares (Levin and Fireman, 2002). A study by Nielsen T.A. et al. (2010) found differences in inhibition and ineffectiveness of dreamed actions and higher levels of anxiety when rating their dreams between nightmare sufferers and healthy controls. Another study focusing on dream content found that interpersonal conflict was one of the most frequent themes of both bad dreams and nightmares (Robert and Zadra, 2014). Sleep disturbance in general increases a sense of loss of control. Increased sleep effort, which typically accompanies sleep disturbance as a maladaptive response to fall asleep, interferes with sleep and exacerbates the feeling of losing control (Espie, 2002). One recent qualitative study identified three superordinate themes associated with insomnia, a sleep disorder highly comorbid with nightmares, and found that “feeling isolated and like an outsider” was a strong theme (Kyle et al., 2010). Thus, it is reasonable to assume that individuals who report sleep disturbance experience interpersonal ineffectiveness. Levin and Nielsen (2007) proposed possible gender differences in emotional brain processes. Females are more inclined to report negative emotional tone of dreams, and nightmare distress is also higher in females compared to males even after controlling for nightmare frequency (Schredl et al., 2014). This suggests that nightmares may especially have a stronger effect for females compared to males.

Although nightmares may generate feelings of ineffectiveness which in turn may lead to suicidal ideation, few studies have examined this relationship in an interpersonal context. Impaired interpersonal functioning and low social support are strong risk factors for suicidality (Rubenowitz et al., 2001; Nickel et al., 2006), and in recent years, the Interpersonal Theory (IPT) of suicide has received wide empirical support. IPT postulates that feelings of interpersonal ineffectiveness, such as perceived interpersonal burdensomeness and thwarted belongingness, accompanied by the acquired capability to die by suicide, predict suicidality (Joiner, 2009). Utilizing IPT as a framework may help explain the association between nightmares and suicidality when considering interpersonal factors. For example, an individual who is suffering from nightmares may feel unable to escape the distress and impairment caused by the nightmare, which could lead to feelings of ineffectiveness, amplifying the perception that they are a burden to those around them. Nightmares may affect interpersonal functioning through emotion dysregulation, increased hyperarousal, or impaired executive functioning from poor sleep quality. Recently, Nadorff et al. (2014) found that nightmares were related to suicide risk and suicide attempts independent of factors of IPT, even after controlling for depressive symptoms. However, their study did not investigate interpersonal factors of IPT as a mediator in explaining the mechanism by which nightmares confer to greater risk for suicidal ideation.

In the current study, we hypothesized that nightmare distress would affect suicidal ideation through interpersonal factors, utilizing IPT as a framework. Nightmare distress can be defined as the negative emotional effects of nightmares in various domains of one’s life. The aim of current study was to (1) investigate the correlations between nightmare distress, suicidal ideation, and interpersonal variables, including thwarted belongingness and perceived burdensomeness; (2) examine if the interpersonal variables of IPT (thwarted belongingness and perceived burdensomeness) act as mediators in the relationship between nightmares and suicidal ideation; and (3) examine whether gender moderated the mediators (thwarted belongingness and perceived burdensomeness) in the relationship between nightmares and suicidal ideation.

## Materials and Methods

### Participants

The current study used a subset of individuals from a larger study that was conducted between March and June in 2015. The original sample consisted of 539 college students taking psychology courses and were recruited from universities in Seoul and Daejeon, Korea. Among this sample, we selected 301 participants who answered “yes” to experiencing nightmares in the past year, which was defined as “an unpleasant and vivid dream that awakens the dreamer from sleep” in this study. All participants completed a questionnaire that included a probe question asking about their experience with nightmares. If the probe question was not positive, the participant was asked to neglect items of the Nightmare Distress Scale. Only the participants who endorsed having nightmares (*n* = 301) were asked to complete the Nightmare Distress Scale, which was the main questionnaire for this study.

### Procedure

Participants completed each of the following measures as part of a larger study. All participants were students in taking a psychology course, and were given the option to participate in a psychology experiment or write a short review paper on a research article for course credit. All participants provided informed consent prior to participation and were compensated with course credit for participating in the study. All questionnaires were completed under supervision of an experimenter to avoid introducing social desirability bias. This research was approved by the Sungshin Women’s University Institutional Review Board.

### Material

All questionnaires below were translated from English to Korean, and then back-translated from Korean to English and compared with the original version by an independent translator who was bilingual and a native English speaker. The participants administrated the Nightmare Distress Questionnaire (NDQ) (Belicki, 1992), the Interpersonal Needs Questionnaire (INQ) and the Depressive Symptoms Inventory – Suicidality Subscale (DSI-SS) (Metalsky and Joiner, 1997; Van Orden et al., 2012).

#### Nightmare Distress Questionnaire

The NDQ is a 13-item self-report questionnaire that assesses the degree of waking distress, negative cognitions, and psychological distress during participants’ wake state as a result of nightmares (Belicki, 1992). Each item is scored on a 5-point Likert scale (score range 0–52). Higher scores reflect greater degree of nightmare distress. Internal consistency was acceptable in our sample (α = 0.84).

#### Interpersonal Needs Questionnaire

The INQ is a 15-item questionnaire designed to assess the desire for suicide based on the IPT of suicidal behavior (Van Orden et al., 2005), which consisted of two subscales including the thwarted belongingness and perceived burdensomeness (Van Orden et al., 2012). The thwarted belongingness subscale consists of nine items and the perceived burdensomeness subscale is comprised of six items. Responses are rated utilizing a 5-point scale ranging from 1 (Not at all true for me) to 5 (Very true for me), with scores ranging from 9 to 45 for thwarted belongingness and 6 to 30 for perceived burdensomeness. Positive items are reverse coded, such that higher scores indicate higher levels of thwarted belongingness and of perceived burdensomeness. The thwarted belongingness sub scale and the perceived burdensomeness sub scale demonstrated good internal consistency in the present study (α = 0.88 and α = 0.91, respectively).

#### Depressive Symptoms Inventory – Suicidality Subscale

The DSI-SS is a 4-item self-report measure designed to measure the presence and intensity of suicidal thoughts, plans, and urges. Each item consists of a group of statements ranging from 0 to 3, with higher scores reflecting more severe suicidality (score range 0–12) (Metalsky and Joiner, 1997). Participants are asked to choose the statement that best explains their experiences within the past 2 weeks. Previous research has found the DSI-SS to have strong psychometric properties and internal consistency in the current sample was high (α = 0.93) (Metalsky and Joiner, 1997; Joiner et al., 2002; Ribeiro et al., 2012).

### Statistical Analyses

**FIGURE 1:**
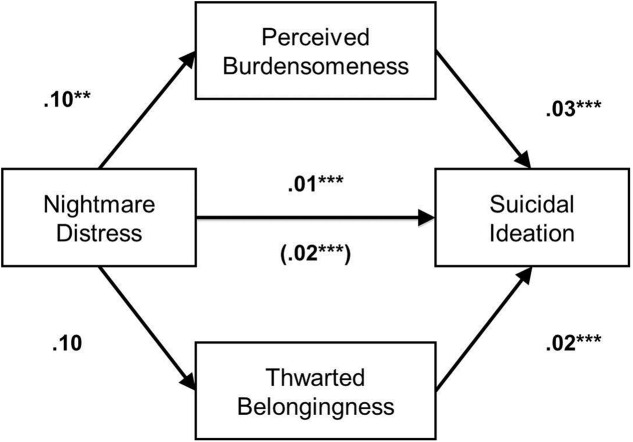
**Unstandardized path coefficients are presented with standard errors in parentheses.** The indirect effect of nightmare distress on suicidal ideation through perceived burdensomeness was significant (Bootstrapped 95% CI ranged from 0.0009 and 0.0099), but not thwarted belongingness (Bootstrapped 95% CI ranged from 0 and 0.0074). ^∗∗^*p* < 0.01, ^∗∗∗^*p* < 0.001.

The current study focused on the mediating effect between nightmare distress and suicidal ideation through interpersonal needs, including thwarted belongingness and perceived burdensomeness. The residual of the dependent variable, suicidal ideation (DSI-SS), violated normality assumptions (Kolmogorov–Smirnov index = 0.39, *p* < 0.001). Thus we used natural log transformation to correct for normality, however, it still violated normality assumptions (Kolmogorov–Smirnov index = 0.41, *p* < 0.001). To examine the hypothesis that nightmare distress is related with suicidal ideation via perceived burdensomeness and thwarted belongingness, multiple mediation analyses were conducted for total NDQ score entered as a predictor of the INQ subscales’ scores, and DSI-SS total score. Unstandardized regression coefficients are presented in **Figure 1**. The bootstrap technique recommended by Shrout and Bolger (2002) was used to test for the mediating effects of thwarted belongingness and perceived burdensomeness on the relationship between nightmare distress and suicidal ideation. Multiple mediation analyses were conducted using the PROCESS macro for Statistical Package for the Social Science, following procedures recommended by Hayes (2013). The indirect effects of thwarted belongingness and perceived burdensomeness on the relationship between nightmare distress and suicidal ideation were evaluated using a bootstrapping resampling procedure: 5,000 bootstrapped samples were drawn from the data, and bias-corrected 95% confidence intervals were used to estimate the indirect effects of each of the resampled datasets (Shrout and Bolger, 2002; Hayes, 2013). Our analysis used bootstrap percentile confidence intervals to infer the observed significance level of the effects. If the confidence interval failed to include 0, the effect was deemed significant (Gardner and Altman, 1986).

## Results

### Demographic Information

The mean age of the sample was 21.87 ± 2.17 (range 18–29), with 78.1% of the sample being female (*n* = 235). The most common living arrangements were living with parents (51.8%), followed by living alone (25.9%), living with friends (10.3%), a relative (2.3%), a partner (1%), or other (8.6%).

### Correlations between Nightmare Distress, Interpersonal Needs, and Suicidal Ideation

Means, standard deviations, and correlations between all study variables are presented in **Table 1**.

**Table 1:** Means, standard deviations, and correlations between study variables.

Variables	1	2	3	4	5	Mean	*SD*	Range
(l) NDQ	–					7.74	7.00	0–52
(2) INQ_total	0.15^∗∗^	–				31.16	9.34	15–75
(3) INQ TB	0.10	0.92^∗∗^	–			22.26	6.54	9–45
(4) INQ PB	0.17^∗∗^	0.79^∗∗^	0.49^∗∗^	–		8.89	4.16	6–30
(5) DSI-SS	0.24^∗∗^	0.43^∗∗^	0.37^∗∗^	0.38^∗∗^	–	1.16	1.93	0–12

*NDQ, Nightmare Distress Questionnaire; INQ, Interpersonal Needs Questionnaire;INQ TB, Interpersonal Needs Questionnaire Thwarted Belongingness; INQ PB, Interpersonal Needs Questionnaire Perceived Burdensomeness; DSI-SS, Depressive Symptom Inventory – Suicidality Subscale score. ^∗∗^p < 0.01.*

### Multiple Mediation Analysis between Nightmare Distress and Suicidal Ideation

**Figure 1** presents the path coefficients from the bootstrapped regression and mediation analyses for the effects of nightmare distress on suicidal ideation through perceived burdensomeness and thwarted belongingness. For analyses examining the relationship between nightmare distress and suicidal ideation, controlling for perceived burdensomeness and thwarted belongingness, the overall regression model explained a significant portion of the variance in suicidal ideation [*R*^2^ = 0.22, *F*(3,297) = 28.28, *p* < 0.001]. Nightmare distress significantly predicted perceived burdensomeness (*B* = 0.10, *SE* = 0.03, *p* = 0.003), and perceived burdensomeness significantly predicted suicidal ideation (*B* = 0.03, *SE* = 0.01, *p* = 0.0001). Although thwarted belongingness was not predicted significantly by nightmare distress (*B* = 0.10, *SE* = 0.05, *p* = 0.06), perceived burdensomeness significantly predicted suicidal ideation (*B* = 0.02, *SE* = 0.006, *p* = 0.0001). The direct effects of nightmare distress on suicidal ideation remained significant after accounting for the effects of perceived burdensomeness and thwarted belongingness (*B* = 0.01, *SE* = 0.005, *p* = 0.0006). The total indirect effect of both mediators on the relationship between nightmare distress and suicidal ideation was estimated to be between 0.0015 and 0.0134 (Bootstrapped 95% CI), indicating significance. The indirect effect of perceived burdensomeness on the relationship between nightmare distress and suicidal ideation was estimated to be between 0.0009 and 0.0099 (Bootstrapped 95% CI), indicating significance.

We also explored gender as a moderator the IPT variables (perceived burdensomeness and thwarted belongingness) in the relationship between nightmare distress and suicidal ideation. We found that gender was a significant moderator for perceived burdensomeness (Index = 0.0083, *SE* = 0.0047, Bootstrapped 95% CI [0.0013, 0.0197]). Thus, perceived burdensomeness was a significant mediator in the relationship between nightmare distress and suicidal ideation in females only, but not males. Gender was not a significant moderator for thwarted belongingness (Index = 0.0056, *SE* = 0.0039, Bootstrapped 95% CI [-0.0004, 0.0154]).

## Discussion

This study provided additional evidence of the important role of interpersonal factors in the relationship between nightmares and suicidal ideation. Among individuals who experienced nightmares, nightmare distress, as measured by the NDQ, was strongly associated with suicidal ideation, as measured by the DSI-SS. Nightmare distress was significantly associated with the perceived burdensomeness subscale, but not the thwarted belongingness subscale. Thus, we found evidence that nightmare distress affects suicidal ideation through perceived burdensomeness.

Nightmare frequency has been associated with suicidality in many studies with various clinical and non-clinical populations (Sjostrom et al., 2007, 2009; Nadorff et al., 2011, 2014); however, relatively few studies have examined underlying mechanisms of this relationship. Although one previous study found that nightmares predict suicidality even when controlling for interpersonal needs (Nadorff et al., 2014), we further examined this relationship to show that perceived interpersonal burden partially mediated the relationship. Although it is unclear why nightmares have this effect (a point that we take up next), these results indicate that interpersonal factors such as perceived interpersonal burdensomeness are important to consider in the relationship between nightmares and suicidal ideation.

### Nightmares and Emotional Dysregulation

One possible way in which nightmare distress leads to unmet interpersonal needs is through disruption of emotional regulation. Many studies have shown a link between sleep in general and a failure to regulate one’s emotions with a few studies showing the role of nightmares specifically creating this effect (Levin and Nielsen, 2009). One study has shown that loss of REM sleep in particular, which is the typical sleep state when awakened from nightmares, hinders one’s adaptation to stressful situations (Greenberg et al., 1972). Emotion dysregulation has been shown to lead to unmet interpersonal needs in general (Slee et al., 2008), as well as both of the individual components of interpersonal needs (Anestis et al., 2011).

### Nightmares and Hyperarousal

Additionally, it has been proposed that nightmares may be a key indicator of hyperarousal, which is an important indicator of acute suicide risk (Ribeiro et al., 2013). Nightmares by definition awaken the dreamer from the unpleasant dream, disrupting sleep continuity, which can result in arousal and sleep-related anxiety (Li et al., 2010). This may further develop into insomnia, which is a condition characterized by high levels of cognitive and physiological hyperarousal.

Hyperarousal has consistently been shown to lead to various sleep problems, such as insomnia and frequent awakenings during the night, as well as nightmares in those with PTSD (Passie et al., 2012). Nightmares initiate a cascade of mental and physical hyperarousal symptoms, which are caused by threats within the disturbing dreams (Krakow and Zadra, 2006). A meta-analysis by McCall and Black has shown that hyperarousal might mediate the relationship between nightmares and suicidality (McCall and Black, 2013). It is hypothesized that hyperarousal might lead to suicide by increasing sensitivity to negative emotional environmental cues or threat cues in the waking state (Cukrowicz et al., 2006). This could potentially explain why those with more nightmare distress have trouble regulating their emotions and lead to having unmet interpersonal needs.

### Nightmares and Impaired Executive Functioning

Another link between nightmares and unmet interpersonal needs is decreased executive functioning. The link between poor sleep quality and decreased executive functioning has been well established (Thomas et al., 2000), however, little research has been done looking specifically at nightmare distress. Nightmares may also cause sleep deprivation, which may subsequently affect executive functioning (Nielsen T. et al., 2010). Individuals with nightmares frequently deliberately avoid initiating sleep or the re-onset of sleep in the middle of the night due to fear of having another nightmare, which may curtail sleep duration and lead to prolonged sleep deprivation (Krakow et al., 1995).

The relationship between executive functioning and suicidal ideation has not focused on interpersonal needs, but it has been shown across populations that those with high levels of suicidal ideation suffer from decreased executive functioning (Dombrovski et al., 2008). It is possible that decreased executive functioning might play a role in relating nightmare distress to suicidal ideation, though more research is needed to examine the link between nightmares and perceived burdensomeness.

### Perceived Burdensomeness as the Sole Mediator between Nightmares and Suicidal Ideation

One interesting finding in our study was that nightmare distress did not lead to suicidal ideation through thwarted belongingness, but solely through perceived burdensomeness. One possible explanation is that those who experience high levels of distress from nightmares feel less control over themselves and ineffective during sleep, which would lead to greater feelings of burdensomeness (Joiner, 2009).

A second possible explanation could be that the events that happen after having nightmares, in addition to the nightmares themselves, lead to people feeling more like a burden yet more connected to others. Clinically, many individuals who suffer from nightmares report that their significant other comforts them after being awakened from a nightmare. Such incidents may increase a sense of perceived burden, but increase a greater feeling of interpersonal connection with a bed partner. Thus, we speculate that the overall effect on thwarted belongingness would be negated by the events occurring after the nightmare; however, this explanation would warrant further research.

Although all of these could potentially explain why nightmares affect perceived burdensomeness and not thwarted belongingness in our study, further research is needed to further delve into the topic. One previous study by Nadorff et al. (2014) found that nightmares predicted suicide risk above and beyond perceived burdensomeness and thwarted belongingness. In our study, we build upon their results by using mediation and moderation analyses. While there are some differences in methodology between these two studies, such as use of different questionnaires and using two culturally different populations, both studies point to the importance of the role of interpersonal needs in the relationship between nightmares and suicidal ideation.

### Clinical Implications

This study provided a further explanation of why nightmare distress leads to suicidal ideation. Although it was done with a non-clinical population, there are numerous potential clinical implications. However, very little research has been done looking into how treating nightmares should progress with suicidal patients, as it may be thought of as a lower priority part of treatment. This study shows that it is a very pervasive problem and nightmare treatment within a suicidal population should be more extensively studied.

In particular, this study shows the importance of addressing problems with nightmares when discussing treatment with suicidal patients, especially when a patient presents with high levels of perceived burdensomeness. It also provides further reasons for use of pharmacological treatments (i.e., prazosin) and non-pharmacological treatments (i.e., imagery rehearsal therapy, IRT) to treat patients with high levels of nightmare distress and are at risk for developing suicidal ideation. Unfortunately neither IRT nor Prazosin have had sufficient research on their efficacy with suicidal patients, but they show promise for future studies. Overall it seems that treatment of nightmares might ameliorate suicidal ideation in multiple ways.

### Limitations

There were a few limitations to the current study that future research should consider. Considering that this study was conducted with a young, non-clinical population, these findings may be different in older or clinical populations. Additionally, we also did not look at mediators outside of interpersonal needs, which could help clarify a full mediation model between nightmare distress and suicidal ideation. Some potential mediators for future research to examine would be the acquired capability for suicide, hopelessness, and anxiety sensitivity. We also did not consider potential confounders such as depression level or other demographic factors, which may have simplified an otherwise complex phenomenon. Another limitation to the study was that the majority of our sample was female, which may have affected the results. For example, tracking menstrual phase in females may be an important factor to consider, as well as use of contraceptives. In addition, the current study used a cross-sectional design, so we were unable to establish a causal relationship between nightmares and interpersonal needs (perceived burdensomeness). Future studies should focus on longitudinal data to uncover the temporal relation of interpersonal factors to nightmare phenomena. Finally, we did not control for the timing of when the questionnaires were completed. Past research indicates that circadian off-peak times of day are associated with greater negativity, and may have affected the results (Tucker et al., 2012). Hopefully our study will lead to further research that will help to further explain to connection between nightmares and suicidal ideation in order to help improve treatment for suicidal ideation.

## Author Contributions

SS and RL contributed to the conception and design of the work and for data acquisition. SS, MS, RL, and TJ interpreted the data and SS and RL did the analyses. SS, MS, RL, and TJ drafted and revised the manuscript. All authors approved of the final version of the manuscript.

## Conflict of Interest Statement

The authors declare that the research was conducted in the absence of any commercial or financial relationships that could be construed as a potential conflict of interest.
